# Novel MeCP2 Isoform-Specific Antibody Reveals the Endogenous MeCP2E1 Expression in Murine Brain, Primary Neurons and Astrocytes

**DOI:** 10.1371/journal.pone.0049763

**Published:** 2012-11-19

**Authors:** Robby M. Zachariah, Carl O. Olson, Chinelo Ezeonwuka, Mojgan Rastegar

**Affiliations:** Regenerative Medicine Program, Department of Biochemistry and Medical Genetics, Faculty of Medicine, University of Manitoba, Winnipeg, Manitoba, Canada; University of Insubria, Italy

## Abstract

Rett Syndrome (RTT) is a severe neurological disorder in young females, and is caused by mutations in the X-linked *MECP2* gene. *MECP2*/*Mecp2* gene encodes for two protein isoforms; MeCP2E1 and MeCP2E2 that are identical except for the N-terminus region of the protein. In brain, *MECP2E1* transcripts are 10X higher, and MeCP2E1 is suggested to be the relevant isoform for RTT. However, due to the unavailability of MeCP2 isoform-specific antibodies, the endogenous expression pattern of MeCP2E1 is unknown. To gain insight into the expression of MeCP2E1 in brain, we have developed an anti-MeCP2E1 antibody and validated its specificity in cells exogenously expressing individual MeCP2 isoforms. This antibody does not show any cross-reactivity with MeCP2E2 and detects endogenous MeCP2E1 in mice brain, with no signal in *Mecp2^tm1.1Bird^* y/− null mice. Additionally, we show the endogenous MeCP2E1 expression throughout different brain regions in adult mice, and demonstrate its highest expression in the brain cortex. Our results also indicate that MeCP2E1 is highly expressed in primary neurons, as compared to primary astrocytes. This is the first report of the endogenous MeCP2E1 expression at the protein levels, providing novel avenues for understanding different aspects of MeCP2 function.

## Introduction

MeCP2 (Methyl CpG Binding Protein 2) was discovered in 1992, as a nuclear protein that binds to methylated DNA [1]. *De novo* mutations in the X-linked *MECP2* gene are associated with more than 90% of reported Rett Syndrome (RTT) cases [2]. RTT is a severe neurological disorder primarily affecting young females with an incidence of 1 in 10,000 live births [3]. RTT patients are mostly asymptomatic up to 6–18 months of age, but start to display impaired locomotor skills, stereotypic hand movements, seizures, abnormal breathing, anxiety and autism [4], [5]. In addition to RTT, *MECP2* mutations have also been detected in patients with classical autism, X-linked mental retardation, Angelman’s syndrome, and severe neonatal encephalopathy [6]–[9].

**Figure 1:**
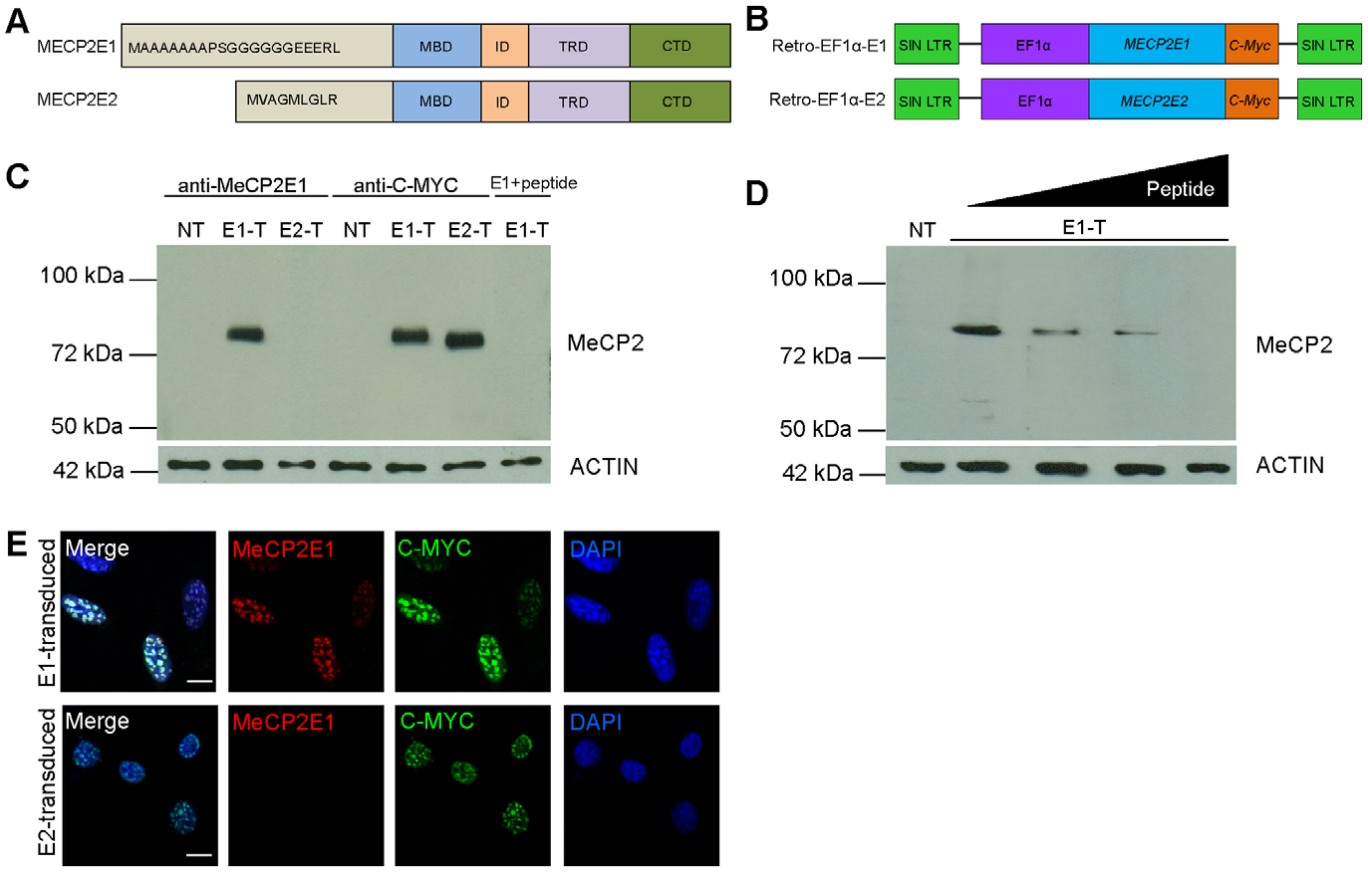
Validation of the newly developed anti-MeCP2E1 antibody. **A)** Schematics of MeCP2 isoforms with known functional domains. The difference in the initial amino acids of the N-terminus is highlighted. **B)** Schematics of the previously reported *MECP2E1* (Retro-EF1α-E1) and *MECP2E2* (Retro-EF1α-E2) retroviral vectors that were used for transfections (C-D) and transductions (E). **C)** Western blot experiments with Phoenix cell extracts from control non-transfected (NT), *MECP2E1* transfected (E1-T), *MECP2E2* transfected (E2-T), and *MECP2E1* with peptide competition. Anti-MYC labelling was used as a positive control and ACTIN was used as a loading control. **D)** Western blot experiments with Phoenix cell extracts from non-transfected cells (NT), and MECP2E1 transfected cells (E1-T), probed with the anti-MeCP2E1 antibody after pre-incubation with increasing concentrations of peptide (0%, 0.1%, 1%, and 5%, of peptide as compared to the amount of antibody used). **E)** Immunofluorescence staining of NIH3T3 cells transduced with *MECP2E1* (top row) or *MECP2E2* (bottom row), with the anti-MeCP2E1 and an anti-C-MYC antibody are shown. DAPI signals are shown in blue. Note that the signals in both transduced cells are detectable with anti-C-MYC, but only transduced cells with *MECP2E1* show positive signals when incubated with the anti-MeCP2E1 antibody. Scale bars represent 10 µm. MBD: methyl binding domain, ID: intervening domain, TRD: transcriptional repression domain, CTD: C-terminal domain.

Alternative splicing of the *Mecp2/MECP2* gene leads to the generation of two protein isoforms, MeCP2E1 (previously called MeCP2B or MeCP2α) and MeCP2E2 (previously called MeCP2A or MeCP2β) [10], [11]. MeCP2 protein isoforms differ only in their N-terminal sequences, sharing the same functional Methyl Binding Domain (MBD) and Transcriptional Repression Domain (TRD) (Fig. 1A). This high degree of similarity between the two MeCP2 isoforms suggests that their functional properties might overlap considerably. However, selective disruption of *Mecp2E2* in mice does not result in the development of RTT phenotypes, which have been observed in mice models where both isoforms are disrupted [12]–[14], indicating that MeCP2E2 is dispensable for RTT pathology [15]. Accordingly, *MECP2E1*-specific mutations are sufficient to cause RTT [16], [17], while no *MECP2E2*-specific mutation has been linked to RTT [18], shifting the spotlight to MeCP2E1 as the more relevant MeCP2 isoform for RTT. In brain, *Mecp2/MECP2* isoforms show differential expression with 10X higher expression of the *MECP*2*E1* [10], [19]. Whether or not MeCP2E1 protein levels follow the transcript expression in brain is unknown, due to the lack of any available MeCP2 isoform-specific antibodies.

Currently, RTT has no effective treatment, however independent groups have shown that reactivation of the *Mecp2* gene after the onset of RTT phenotypes in mice, partially rescues physiological and anatomical abnormalities [20]–[22]. This suggests that gene therapy delivery of *MECP2* into affected neurons may improve RTT symptoms. We reported the first preclinical *MECP2E1* retroviral and lentiviral gene therapy vectors [23]. We also showed the functional rescue potential of *MECP*2*E1* gene therapy vectors in recovering aberrant neuronal dendrite branching of *Mecp2* deficient neurons [23]. In mice, *Mecp2* deficiency in neurons is sufficient to cause RTT-like phenotype [13], and cell type-specific depletion in different brain regions are associated with particular phenotypes [13], [24]–[28]. For future gene therapy delivery of *MECP2E1* to rescue particular phenotypes, a comprehensive knowledge of MeCP2E1 protein expression in brain is required.

In the present study, we report the generation and validation of an isoform-specific anti-MeCP2E1 antibody. We demonstrate the specificity of this antibody in *MECP2E1* overexpressing cells, using western blot (WB) and immunofluorescent (IF) techniques and confirm the absence of any cross-reactivity with MeCP2E2. We further show that our newly developed anti-MeCP2E1 antibody recognizes the endogenous murine MeCP2E1 by WB and immunohistochemistry (IHC) assays and investigate the corresponding protein expression in different brain regions of adult murine brain. Subsequently, we report that MeCP2E1 exhibits higher expression in primary neurons as compared to primary astrocytes. Our newly developed anti-MeCP2E1 antibody is a novel tool for comprehensive research studies on MeCP2E1, presenting new avenues of research into MeCP2E1 function and its crucial role in the maintenance of normal brain function and development.

## Materials and Methods

### Ethics Statement

Experiments were conducted in accordance with the standards of the Canadian Council on Animal Care with the approval of the Office of Research Ethics of the University of Manitoba. All experiments conducted with mice were in accordance with animal experimentation guidelines (University of Manitoba). *MEPC2* knockout mice (*Mecp2^tm1.1Bird^*) were obtained from The Jackson laboratories, USA along with their wild type counterparts. All experimental procedures outlined here, were reviewed and approved (protocol number 09-020/1/2) by the University of Manitoba Bannatyne Campus Protocol Management and Review Committee.

**Figure 2:**
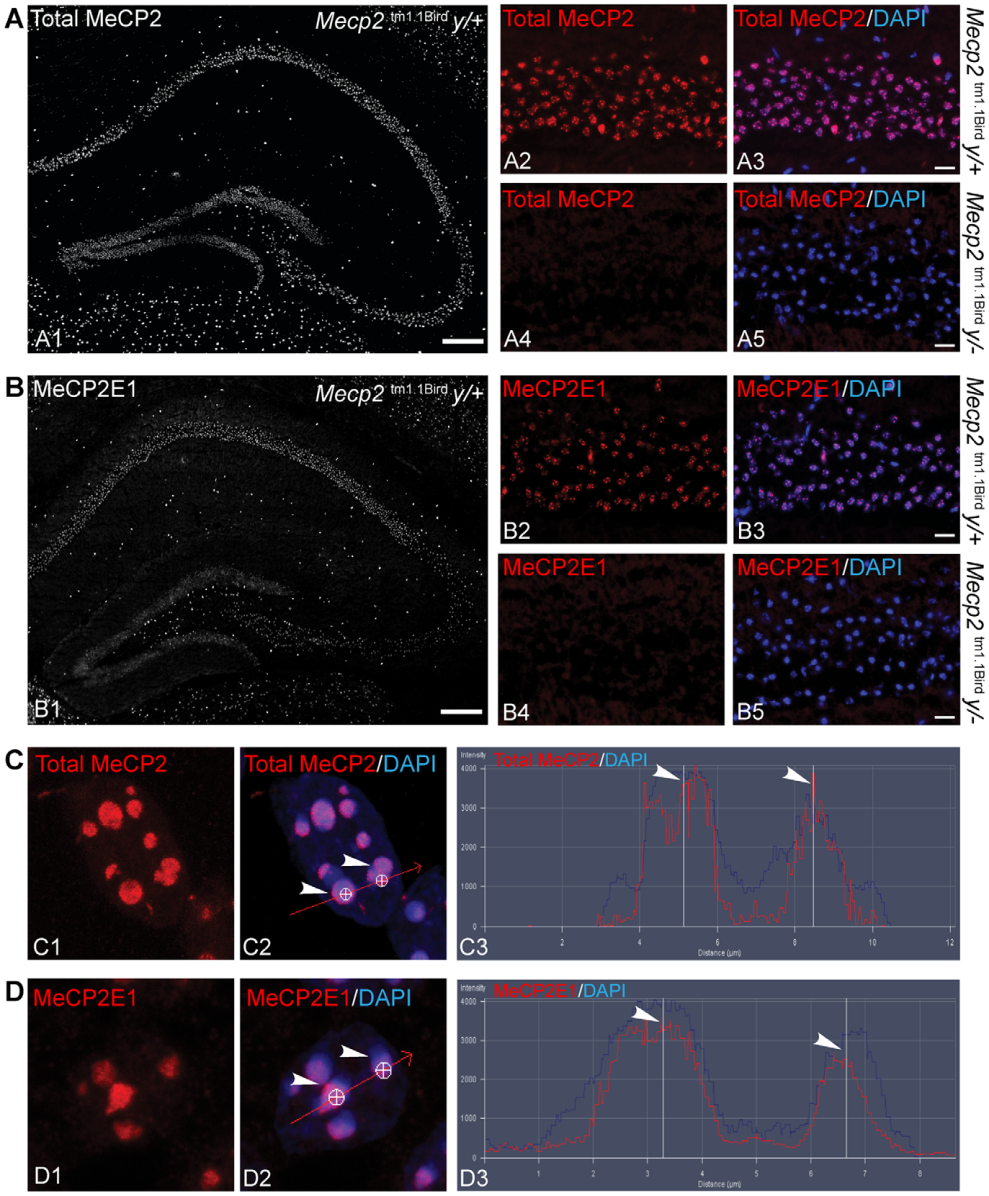
Anti-MeCP2E1 validation by immunohistochemistry and MeCP2E1 detection in adult mouse hippocampus. **A)** A tiled image of MeCP2 immunolabelling in the adult mice hippocampus (A1) is shown. A higher magnification of MeCP2 immunolabelling (A2, red) in hippocampus CA1 region of adult mouse is shown, as well as the merged signals with DAPI signals (blue) in the nuclei (A3). As a negative control for A2–A3, the absence of MeCP2 immunolabelling is shown (A4, red) in the hippocampus CA1 region of *Mecp2^tm1.1Bird^* y/− mouse where DAPI (blue) labelling in nuclei is present and it is shown in the merged image (A5). **B)** A tiled image of MeCP2E1 immunolabelling in the hippocampus (B1) of adult mice brain. Higher magnification of MeCP2E1 immunolabelling (B2, red) in the hippocampus CA1 region of adult mouse brain, and the merge image with DAPI signals (blue) in the nuclei (B3). The absence of MeCP2E1 immunolabelling (B4) in hippocampus CA1 region of adult mouse brain where DAPI signals (blue) in the nuclei is shown in the merged image (B5). **C)** Confocal images of MeCP2 (red) immunolabelling in adult mice hippocampus in the CA1 region (C1), shown to overlap with DAPI (blue) nuclear labelling (C2). Signal intensity profile analysis of C2 in two white circles (shown by white arrows), shows enriched MeCP2 signals localized at the DAPI-rich nuclear regions of cells within the hippocampus CA1 (C3). **D)** Confocal images of MeCP2E1 (red) in wild type adult mouse brain in the hippocampus CA1 region (D1), and overlap with DAPI (blue) nuclear labelling (D2). Signal intensity profile analysis indicates enriched MeCP2E1 (D3) localized at the DAPI-rich regions of hippocampus CA1 nuclei. Scale bars: A2–A5, B2–B5 = 20 µm; A1, B1 = 200 µm.

### MeCP2E1 Antibody Generation

A peptide sequence from the N-terminus of MeCP2E1 isoform (GGGEEERLEEK) that is conserved in murine and human MeCP2 protein was selected as the antigen for polyclonal antibody production in chicken. The IgY molecules were purified from chicken egg yolks and anti-MeCP2E1-specific immunoglobulins were isolated by peptide affinity purification.

**Figure 3:**
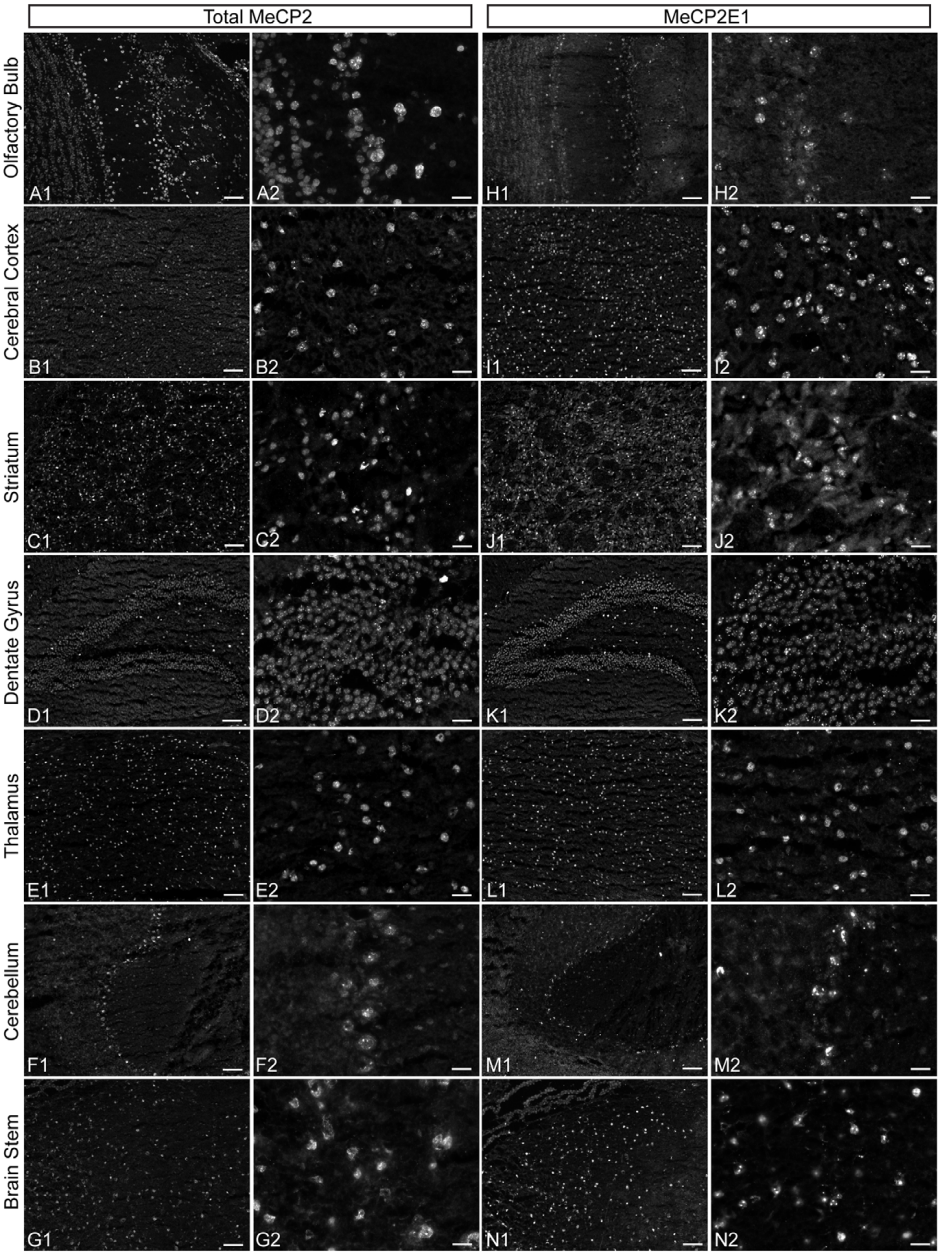
Expression of total MeCP2 and MeCP2E1 in adult murine brain. Expression of total MeCP2 and MeCP2E1, respectively in olfactory bulb (A1, H1), cerebral cortex (B1, I1), striatum (C1, J1), dentate gyrus of hippocampus (D1, K1), thalamus (E1, L1), cerebellum (F1, M1) and brain stem (G1, N1). Higher magnification images for total MeCP2 (A2–G2) and MeCP2E1 (H2–N2) shows their nuclear expression pattern within the various brain regions. Scale bars: A1–G1; H1–N1 = 80 µm, A2–G2; H2–N2 = 20 µm.

### Generation of *MECP2E1/E2* Transfected/Transduced Cells

The construction of retroviral *MECP2E1* and *MECP2E2* vectors with a C-terminal *C-Myc* tag has been described previously [23]. To generate infectious retroviral particles, Retro-EF1α-E1 (expressing *MECP2E1*) and Retro-EF1α-E2 (expressing *MECP2E2*) vectors were transfected into Phoenix retroviral packaging cells [29] as described previously [23]. Culture supernatants containing viral particles were harvested at 48 hours (h) post-transfections. The transfected Phoenix cells were collected and lysed for protein extraction, and the retroviral particles were used to transduce NIH3T3 mouse fibroblasts with the same protocol as described before [23]. The transduced cells were fixed with 4% paraformaldehyde for immunofluorescent studies, 48 h after transduction.

**Figure 4:**
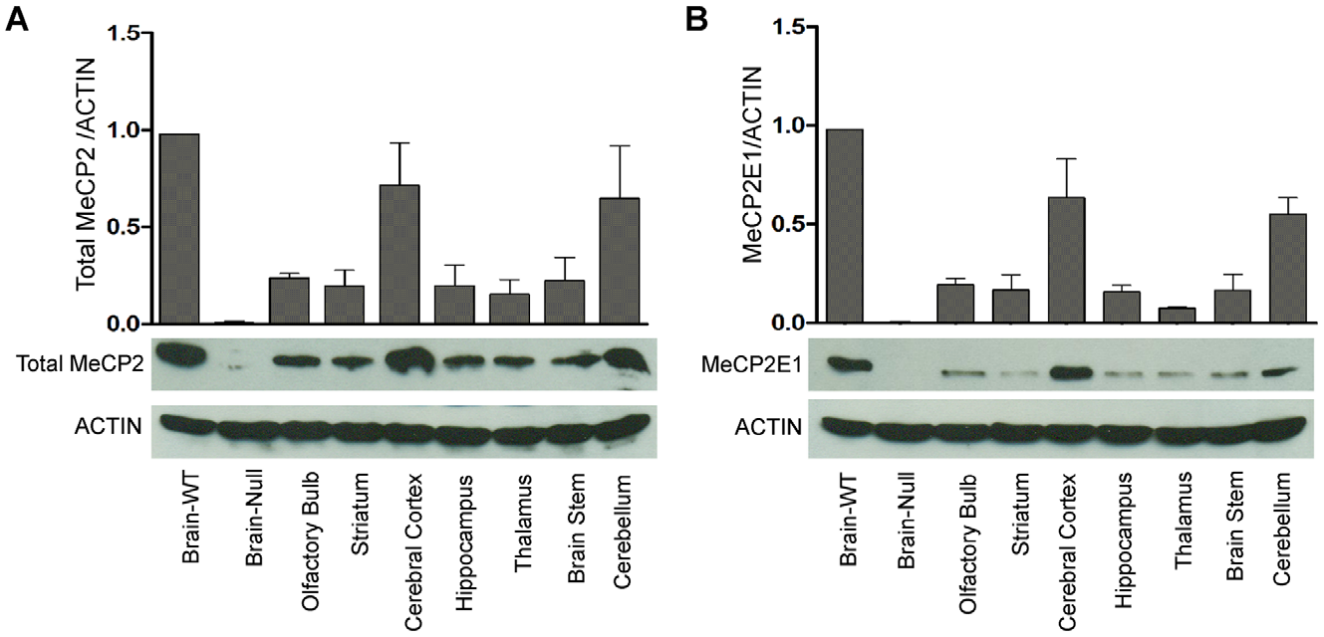
Differential expression of total MeCP2 and MeCP2E1 in adult murine brain regions. Quantification of total MeCP2 (**A**) and MeCP2E1 (**B**) in total cell extracts from the wild type *Mecp2^tm1.1Bird^* y/+ mice whole brain (Brain-WT), olfactory bulb, striatum, cerebral cortex, hippocampus, thalamus, brain stem and cerebellum. *Mecp2^tm1.1Bird^* y/− mice whole brain (Brain-Null) was included as a negative control. Equal loading of protein lysates was verified by probing the same membrane with ACTIN (N = 3±SEM).

### Isolation of Primary Neurons and Astrocytes

Postmitotic cortical neurons were isolated from embryonic day (E) 18 mouse embryos from a CD1 background as described before [23], [30]. Briefly, cerebral cortices were dissected from E18 mouse embryos, dissociated using Papain and triturated with a fire-polished Pasteur pipette. Subsequently, the cells were resuspended in Neurobasal medium with B-27 supplement and plated at a density of 1.2 × 10^5^ cells/ml in poly-lysine coated dishes. After three days, 50% of the media was replaced with fresh medium. Subsequently, media was replenished every 48 h. Cells were lysed/fixed 7 days after seeding for further experiments.

**Figure 5:**
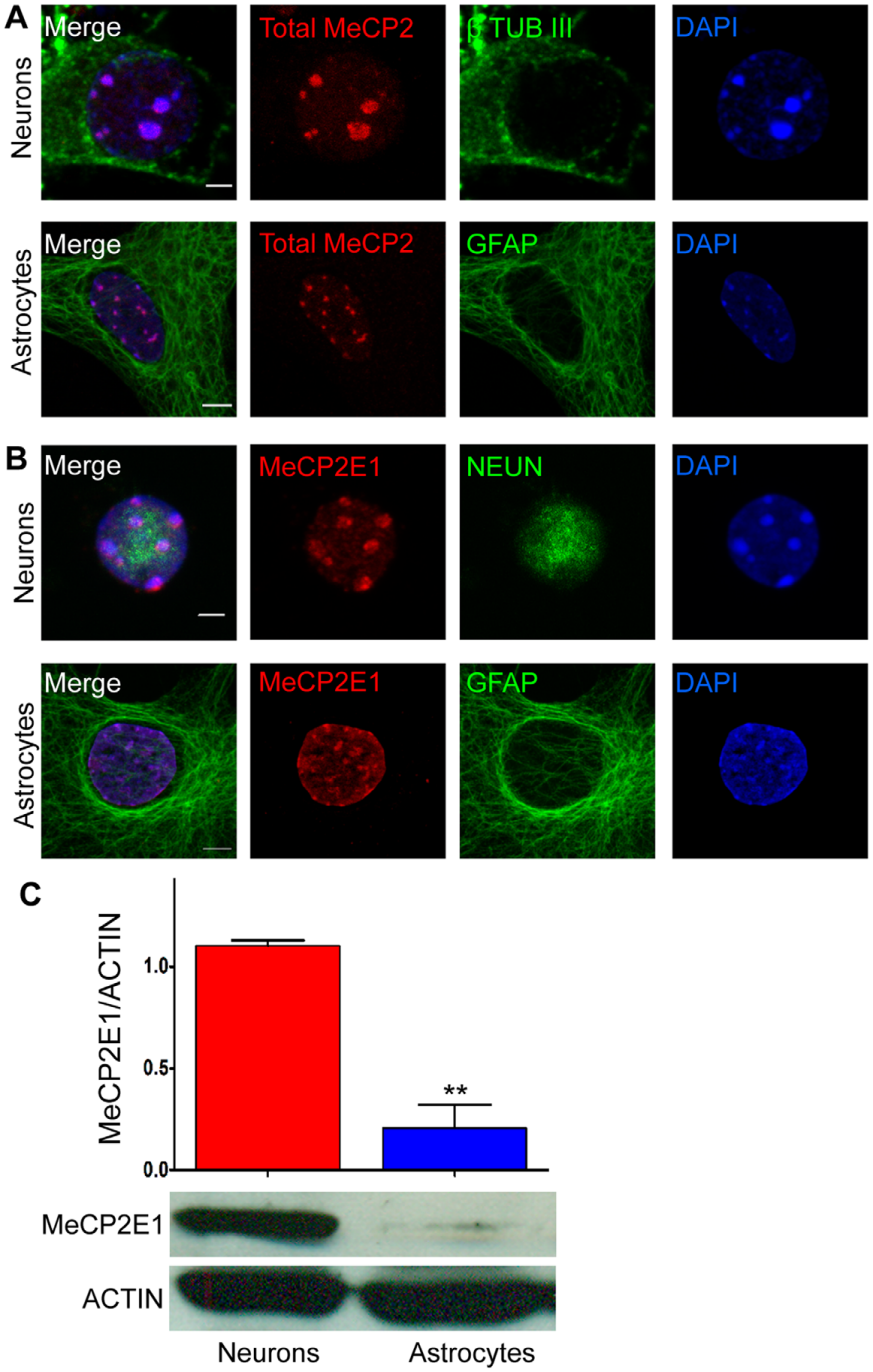
Expression of total MeCP2 and MeCP2E1 in primary neurons and astrocytes. A ) Expression of total MeCP2 in embryonic primary cortical neurons and astrocytes are detected by immunofluorescence labelling. Cells were labelled with β-III tubulin (β TUB III) and GFAP to mark neurons and astrocytes, respectively. **B**) Expression of MeCP2E1 in primary cortical neurons and astrocytes. Cells were labelled with NEUN and GFAP to mark neurons and astrocytes, respectively. Scale bars represent 5 µm. **C**) Western blot analysis of MeCP2E1 levels in primary cortical neurons and astrocytes. The graph depicts the quantification of MeCP2E1 in neurons and astrocytes, p<0.01 (N = 2±SEM).

Primary cortical astrocytes were isolated from E18 mouse embryos from a CD1 background as described [31]. Briefly, cerebral cortices were isolated from E18 mouse embryos and dissociated using Papain. The cells were triturated using narrow-ended pipettes and resuspended in MEM with 10% FBS. Subsequently, the cells were seeded at a density of 2 × 10^5^ cells/ml in poly-lysine coated dishes. Media was replaced every 48 h. Cells were lysed/fixed 14 days after seeding for further experiments.

### Immunohistochemistry, Immunofluorescence and Fluorescent Imaging

For IHC studies, brain tissues were isolated from euthanized mice (C57/BL 6), cut into small blocks and incubated in freshly de-polymerized paraformaldehyde fixative solution (0.16 M sodium phosphate buffer, pH 7.4 with PFA) for 20 minutes (min) and rinsed with cryoprotectant solution (25 mM sodium phosphate buffer, pH 7.4, 10% sucrose, 0.04% NaN3). Subsequently, the tissue blocks were incubated in cryoprotectant at 4°C for approximately 24 h. Cryosections were processed on to gelatinized slides and stored at −20°C. Prior to IHC experiments, slides were air-dried and permeabilized for 20 min with 0.3% Triton X-100 Tris-buffered saline (TBS-Tr) (50 mM Tris-HCl, pH 7.4, containing 1.5% NaCl) solution. The slides were then pre-blocked with 20% normal donkey serum (NDS, Jackson Immunoresearch, 005-000-123) in TBS-Tr and incubated with appropriate primary antibodies diluted in 10% NDS in TBS-Tr overnight at 4°C. Primary antibody incubation was followed by three washes with TBS-Tr. Secondary antibodies in diluted TBS-Tr/10% NDS were applied for 1 h at room temperature, followed by one wash with TBS-Tr and two washes using Tris-HCl buffer (50 mM, pH 7.4). The slides were then mounted on Prolong Gold antifade containing 2 µg/ml 4′,6-diamidino-2-phenylindole (DAPI) (Calbiochem, EMD Millipore, Billerica MA) counter-stain.

For IF studies, cells were fixed with 4% formaldehyde for 10 min on ice and processed as described previously [23]. The coverslips were then slide-mounted with anti-fade medium containing DAPI (0.5 µg/ml). Immunolabelled signals were detected using a Zeiss Axio Observer Z1 inverted microscope and LSM710 Confocal microscope (Carl Zeiss, Canada Ltd, Toronto, ON). Images were obtained using AxioVision 4.8, Zen Blue 2011, Zen Black 2009 and Zen Black 2011 softwares (Carl Zeiss Canada Ltd) and assembled using Adobe Photoshop CS5 and Adobe Illustrator CS5.

### Western Blotting

Total cell extracts were prepared and WB was done as described previously [23], [32]. For WB experiments, 2 µg of total protein extracts from transfected cells or 100 µg of total cell extracts from brain, primary neurons or astrocytes were loaded in each lane and were subjected to WB analysis. All probed membranes were subjected to a second WB with an anti-ACTIN antibody as a loading control. Quantification of detected MeCP2 or MeCP2E1 bands was done with Adobe Photoshop CS5 software and all bands were normalized to ACTIN signals. Student’s t-test was used to analyze the significance of MeCP2 protein levels between samples. For peptide incubation experiments, increasing amounts of peptide antigen (as compared to the antibody concentration) was pre-incubated with the antibody for 3–5 h at 4°C before probing the membrane.

### Antibodies

The following antibodies were used in this study: mouse monoclonal anti-MeCP2 (ab50005, WB-1∶1000; IF-1∶200), rabbit polyclonal anti-H3K9me3 (ab8898, IF-1∶200), rabbit polyclonal anti-H4K20me3 (ab9053, IF-1∶200), mouse monoclonal anti-H3K27me3 (ab6002, IF-1∶200), mouse monoclonal anti-H3K9me2 (ab1220, IF-1∶200) [Abcam]; rabbit polyclonal anti-MeCP2 (07–013, WB-1∶1000, IF-1∶200), mouse monoclonal anti-β-Tubulin III (MAB1637, IF-1∶200), mouse monoclonal anti-NEUN (MAB377, IF-1∶200), chicken polyclonal anti-β-Tubulin III (AB9354, IF-1∶200) [Millipore]; mouse monoclonal anti-C-MYC (A21280, WB-1∶1000, IF-1∶200), mouse monoclonal anti-GFAP (A21282, IF-1∶200) [Molecular Probes]; mouse monoclonal anti-ACTIN (A2228, WB-1∶2500) [Sigma Aldrich].

The following secondary antibodies were used: Gt anti-rabbit Alexa Fluor 488 (A11034, IF-1∶1000), Gt anti-rabbit Alexa Fluor 594 (A11037, IF-1∶1000), Gt anti-chicken Alexa Fluor 594 (A11042, IF-1∶1000) [Molecular Probes]; Gt anti-rabbit Rhodamine Red-X (111-295-144, IF-1∶400), Gt anti-chicken Rhodamine Red-X (103-295-155, IF-1∶400), Peroxidase-Affinipure Gt anti-mouse IgG (115-035-174; WB-1∶7500), Perox-AffiniPure Dnk anti-rabbit IgG (711-035-152; WB-1∶400) [Jackson ImmunoResearch].

## Results

### Generation and Validation of Anti-MeCP2E1 Antibody in vitro

In the present study, we developed an anti-MeCP2E1 isoform-specific antibody to investigate the endogenous expression of MeCP2E1 protein. The polyclonal chicken anti-MeCP2E1 antibody was generated using a synthetic peptide spanning the N-terminal region of MeCP2E1. Specificity of this anti-MeCP2E1 antibody was validated by WB and IF experiments throughout the course of antibody production and after IgY purification. For WB application, the purified antibody was tested using cell extracts from Phoenix cells transfected with either Retro-EF1α-E1 or Retro-EF1α-E2 (Fig. 1B), in parallel to non-transfected control cells. As expected, WB analysis with the anti-MeCP2E1 antibody yielded a specific band at the expected molecular weight (slightly higher than 72 kDa) in MeCP2E1-transfected cells (E1-T, Fig. 1C, lane 2). In contrast, no bands were detected in non-transfected cells (NT, Fig. 1C, lane 1), nor in the transfected cells with *MECP2E2* (E2-T, Fig. 1C, lane 3). Importantly, pre-incubation of the anti-MeCP2E1 antibody with the antigenic peptide used to generate the antibody (peptide competition) eliminated the detected band in the MeCP2E1 transfected cells (Fig. 1C, lane 7). The presence of exogenous MeCP2 in the transduced cells with either Retro-EF1α-E1 or Retro-EF1α-E2 was confirmed by immunolabelling with an anti-C-MYC antibody (Fig. 1C, lanes 5–6), with no detectable signal in non-transfected cells (Fig. 1C, lane 4). The specificity and sensitivity of the newly developed antibody was further verified by pre-incubation of the anti-MeCP2E1 antibody with increasing concentrations of the antigenic peptide before probing the membranes with *MECP2E1* transfected cell lysates (Fig. 1D, lanes 2–5). Non-transfected Phoenix cell lysates were used as a negative control (Fig. 1D, lane 1).

IF staining with the anti-MeCP2E1 antibody revealed the expression of MeCP2 in the DAPI-rich heterochromatic foci within the NIH3T3 cells transduced with *MeCP2E1,* but no signal was detected in the *MECP2E2* transduced cells (Fig. 1E). This indicates that our newly developed anti-MeCP2E1 antibody does not cross-react with the overexpressed MeCP2E2. In both MeCP2E1 and MeCP2E2 overexpressed cells, incubation with an anti-C-MYC antibody resulted in detectable signals indicating that the transduced protein is properly expressed in both cases. The absence of endogenous MeCP2E1 expression was confirmed in the non-transduced NIH3T3 cells using the anti-MeCP2E1 antibody (Fig. S1A). We did not detect any signal in primary omission experiments using Retro-EF1α-E1 transduced cells with the same secondary antibody, as expected (Fig. S1B).

These results demonstrate that the newly generated anti-MeCP2E1 antibody specifically detects MeCP2E1 protein, and shows no cross-reactivity with MeCP2E2.

### The Newly Generated Anti-MeCP2E1 Antibody Shows Specificity in Detecting Endogenous MeCP2E1 in Mice

In order to investigate whether the newly developed anti-MeCP2E1 antibody is capable of detecting the endogenous MeCP2E1, we tested the specificity of this antibody in murine adult brain. Previous studies have reported that MeCP2E1 is highly expressed in murine adult brain [33]. In agreement with these reports, our immunohistochemical experiments using a commercial anti-MeCP2 antibody (recognizing both MeCP2E1 and MeCP2E2 isoforms) detected specific nuclear staining in the murine wild type (WT) hippocampus (Fig. 2; A1–A3). As a negative control, we performed IHC experiments in the null male *Mecp2^tm1.1Bird^* y/− transgenic RTT mouse model, where exons 3 and 4 of *Mecp2* are deleted, and *Mecp2* transcripts and protein are non-detectable [12]. Repeating the IHC experiments in the *Mecp2^tm1.Bird^* y/− null male mice at 4–6 weeks of age, we did not detect any MeCP2 signals (Fig. 2; A4–A5), as expected.

Next, we studied whether under similar conditions we can detect visible signals using our newly developed anti-MeCP2E1 antibody. Immunohistochemical experiments with our anti-MeCP2E1 antibody revealed nuclear MeCP2E1 signals in the adult WT hippocampus (Fig. 2; B1–B3), with no detectable signals in the *Mecp2^tm1.Bird^* y/− null male mice (Fig. 2; B4–B5). Confocal imaging and subsequent signal profile analysis revealed that MeCP2E1 expression has enriched localization at the DAPI-rich heterochromatic regions, similar to what is observed for total MeCP2 (Fig. 2; C1–C3, D1–D3). No signal was observed with negative controls when we used chicken 1 gY instead of polyclonal chicken anti-MeCP2E1 antibody or when we performed anti-MeCP2E1 peptide competition in hippocampal regions under identical experimental conditions (Fig. S2A and S2B).

Taken together, these results indicate that our newly developed anti-MeCP2E1 antibody specifically detects the endogenous murine MeCP2E1 and that MeCP2E1 shows similar nuclear localization compared to the total MeCP2 in the DAPI-rich heterochromatin regions of the nucleus.

### MeCP2E1 is Expressed at Different Levels within Different Regions of Murine Brain

MeCP2 displays highest expression in brain, as compared to other tissues [33]–[35]. Previous studies on *Mecp2E*1 and *Mecp2E2* transcripts have reported variable transcript abundance in a brain region-specific manner [19]. Additionally, it is known that the transcript levels of *Mecp2* and the corresponding protein expression are non-complementary [33]. This highlights the need to determine the expression of MeCP2E1 at the protein level. Therefore, we next examined the expression of MeCP2E1 within various regions of the adult murine brain including olfactory bulb, cerebral cortex, striatum, hippocampus/dentate gyrus, thalamus, brainstem and cerebellum. We initially examined total MeCP2 expression detected by a commercial C-terminal antibody in the above-mentioned regions across the WT adult mice brain. As expected, MeCP2 expression was detectable in all tested brain regions and showed the characteristic nuclear MeCP2 signals (Fig. 3; A1–G1, and A2–G2 with higher magnification). Immunolabelling with the anti-MeCP2E1 antibody demonstrated a broad distribution pattern of endogenous MeCP2E1 across all these studied regions of the murine brain (Fig. 3; H1–N1, and H2–N2 with higher magnification).

Quantitative analysis of total MeCP2 levels within these tested brain regions by WB of total cell extracts demonstrated that the highest levels of total MeCP2 are detected in cerebral cortex and cerebellum as compared to other regions (Fig. 4A). Analysis of MeCP2E1 levels in these regions, revealed a similar pattern (Fig. 4B), suggesting that MeCP2E1 is likely the major MeCP2 isoform expressed in these regions. This is in agreement with previous studies in adult rodent brain that had shown high levels of MeCP2 mRNA expression in cerebral cortex and cerebellum [33], [36].

Taken together, these indicate that MeCP2E1 is predominantly and broadly expressed within the adult murine brain, with differential expression levels in various brain regions.

### MeCP2E1 has Higher Expression in Primary Neurons Compared to Primary Astrocytes

The expression of MeCP2 in astrocytes has been a relatively recent discovery, which has lead to a significant paradigm shift on the contribution of glial cells towards RTT pathophysiology. Re-expression of MeCP2 in astrocytes in RTT mice models mitigates many RTT phenotypes [37]. However the expression of MeCP2 isoforms and their potential role in astrocyte function remain to be determined. Additionally, the expression of MeCP2 protein isoforms at the protein levels in neurons is still unknown. Therefore, we next used our newly developed anti-MeCP2E1 antibody and examined the expression of MeCP2E1 in primary cortical neurons and astrocytes. As expected, we detected the endogenous expression of total MeCP2 in both primary cortical neurons and astrocytes using the C-terminal anti-MeCP2 antibody (Fig. 5A). Previous studies have suggested that MeCP2 expression in primary neurons might vary from diffuse to punctuate staining within the nucleus based on culture conditions [38], [39]. Therefore, we examined the nuclear MeCP2 expression in these primary neurons by confocal co-localization studies compared to constitutive and facultative heterochromatin marks. As shown in supplementary figure 3 (Fig. S3), MeCP2 is primarily co-localized with the two tested constitutive heterochromatin marks (H3K9me3, H4K20me3), but showed minimal overlapping pattern with the facultative heterochromatin marks (H3K27me3, H3K9me2).

Immunofluorescence experiments with our newly generated anti-MeCP2E1 antibody detected endogenous MeCP2E1 expression in both primary neurons and astrocytes with nuclear heterochromatic expression pattern overlapping with DAPI signals (Fig. 5B). Although, this indicates that MeCP2E1 has similar nuclear localization compared to the total MeCP2 in both primary neurons and astrocytes, it does not reflect the protein levels in these two cell types. As a quantitative approach, we examined the total amount of MeCP2E1 in primary neurons and astrocytes by WB analysis and compared it to the ACTIN levels. We found that that MeCP2E1 levels are indeed five times higher in primary neurons as compared to primary astrocytes (Fig. 5 C). This is not surprising, as primary astrocytes are reported to express approximately 25% of MeCP2 levels observed in primary neurons [40].

Taken together, these results indicate that while MeCP2E1 is expressed in both primary cortical neurons and astrocytes, its level of expression is significantly higher in neurons. Our data further indicate that in both primary neurons and astrocytes, MeCP2E1 signals highly overlap with DAPI-rich heterochromatin regions in the nucleus. Additionally, we show that punctuated MeCP2 heterochromatic localization in neurons, shows significant overlap with constitutive heterochromatin marks, but has low overlap with the facultative heterochromatin marks.

## Discussion

In this study, we generated an anti-MeCP2E1 isoform-specific antibody and investigated the endogenous expression of MeCP2E1 within the adult murine brain. Recent studies have demonstrated that both MeCP2 isoforms are capable of rescuing RTT phenotypes upon transgenic expression. Remarkably, MeCP2E1 transgenic expression was sufficient in reversing the majority of RTT phenotypes, even at lower expression levels [41]. These findings are highly significant in terms of future gene therapy strategies for RTT patients. Our previous studies have shown the feasibility of inducing MeCP2 isoform-specific expression for reversing cellular phenotypes of *Mecp2*-deficient neurons such as dendrite branching [23]. However, the endogenous expression pattern of MeCP2E1 has remained undetermined to date, mainly due to the unavailability of any MeCP2E1 isoform-specific antibody. Our newly developed MeCP2E1 antibody provides novel avenues for understanding brain region and/or cell type-specific expression of MeCP2E1, offering vital insights for the efficient design of future gene therapy approaches.

The manifestation of RTT phenotypes at the postnatal stage suggests that MeCP2 plays a critical role in normal brain development [5]. However, recent studies have provided several lines of evidence on the critical requirement of normal MeCP2 expression and function even at the adulthood [42], [43]. Depletion of *Mecp2* in adult mice models induce RTT-like phenotypes and display similar kinetics in terms of symptomatic progression and lethality, as compared to postnatal loss of MeCP2 [43]. Our findings demonstrate the widespread distribution of MeCP2E1 within the adult murine brain. More importantly, our results suggest that MeCP2E1 displays more abundant expression in specific brain regions. The significance of this brain-region specific enrichment of MeCP2E1 expression remains to be elucidated. Comparison of MeCP2E1 cellular localization signals with the expression pattern detected by a C-terminal MeCP2 antibody suggests that both MeCP2 isoforms display similar nuclear heterochromatic expression *in vivo*, in agreement with previous studies [41].

Initial studies on MeCP2 suggested that MeCP2 expression is exclusive to neurons within the developing brain. In 2009, several independent groups provided evidence for MeCP2 expression within glial subtypes [23], [40], [44]. Furthermore, re-expression of *Mecp2* preferentially in astrocytes ameliorated many RTT phenotypes [37]. In the present study, we report that E18-derived mouse primary neurons express MeCP2E1 protein at significantly 5X higher levels than in primary astrocytes. Our results are in agreement with previous observations that demonstrated total MeCP2 expression in astrocytes being approximately 25% of the levels in neurons [40]. Further studies will be required to verify the corresponding levels of MeCP2E2 in neurons and astrocytes. The higher abundance of MeCP2E1 in primary neurons compared to astrocytes suggests that MeCP2E1 might be significantly contributing towards the physiological symptoms associated with Rett syndrome. However, the functional significance of MeCP2 isoforms in neurons and glial cells remains to be elucidated.

In summary, we have successfully generated an anti-MeCP2E1 antibody and for the first time, report the endogenous expression of MeCP2E1 in the adult murine brain. Furthermore, our results demonstrate the differential distribution of MeCP2E1 within various brain regions. We further show that MeCP2E1 is more abundant in neurons as compared to astrocytes. Understanding the endogenous expression of MeCP2E1 will be instrumental in gaining further insights into the pathophysiology of Rett Syndrome.

## Supporting Information

Figure S1 **Controls for MeCP2 overexpression in NIH3T3 cells. A**) Absence of MeCP2 and C-MYC signals in non-transfected NIH3T3 cells. **B**) Absence of signals in primary omission controls with Rhodamine Red and FITC in *MECP2E1* transfected NIH3T3 cells. Images are taken at the same exposure time as in Figure 1E. Scale bars represent 10 µm.
(TIFF)

Figure S2 **Controls to verify the specificity of MeCP2E1 immunolabelling within the adult murine brain. A**) The negative control IgY did not generate any signals in *Mecp2^tm1.1Bird^* y/+ mice. **B**) Pre-incubation of the newly generated anti-MeCP2E1 with the antigenic peptide resulted in absence of specific labelling in *Mecp2^tm1.1Bird^* y/+ mice. Scale bars represent 20 µm.
(TIFF)

Figure S3 **Nuclear localization of MeCP2 and heterochromatin marks in primary neurons. A**) MeCP2 signals in embryonic primary cortical neurons display overlapped signals with constitutive heterochromatin marks; H3K9me3 and H4K20me3. **B**) MeCP2 displays minimal overlap with facultative heterochromatin marks; H3K27me3 and H3K9me2. Scale bars represent 2 µm.
(TIFF)
