# Supplementary figures and images for: Novel MeCP2 Isoform-Specific Antibody Reveals the Endogenous MeCP2E1 Expression in Murine Brain, Primary Neurons and Astrocytes
Source: PLoS One. 2012 Nov 19;7(11):e49763. doi: 10.1371/journal.pone.0049763 (PMC3501454; doi:10.1371/journal.pone.0049763)

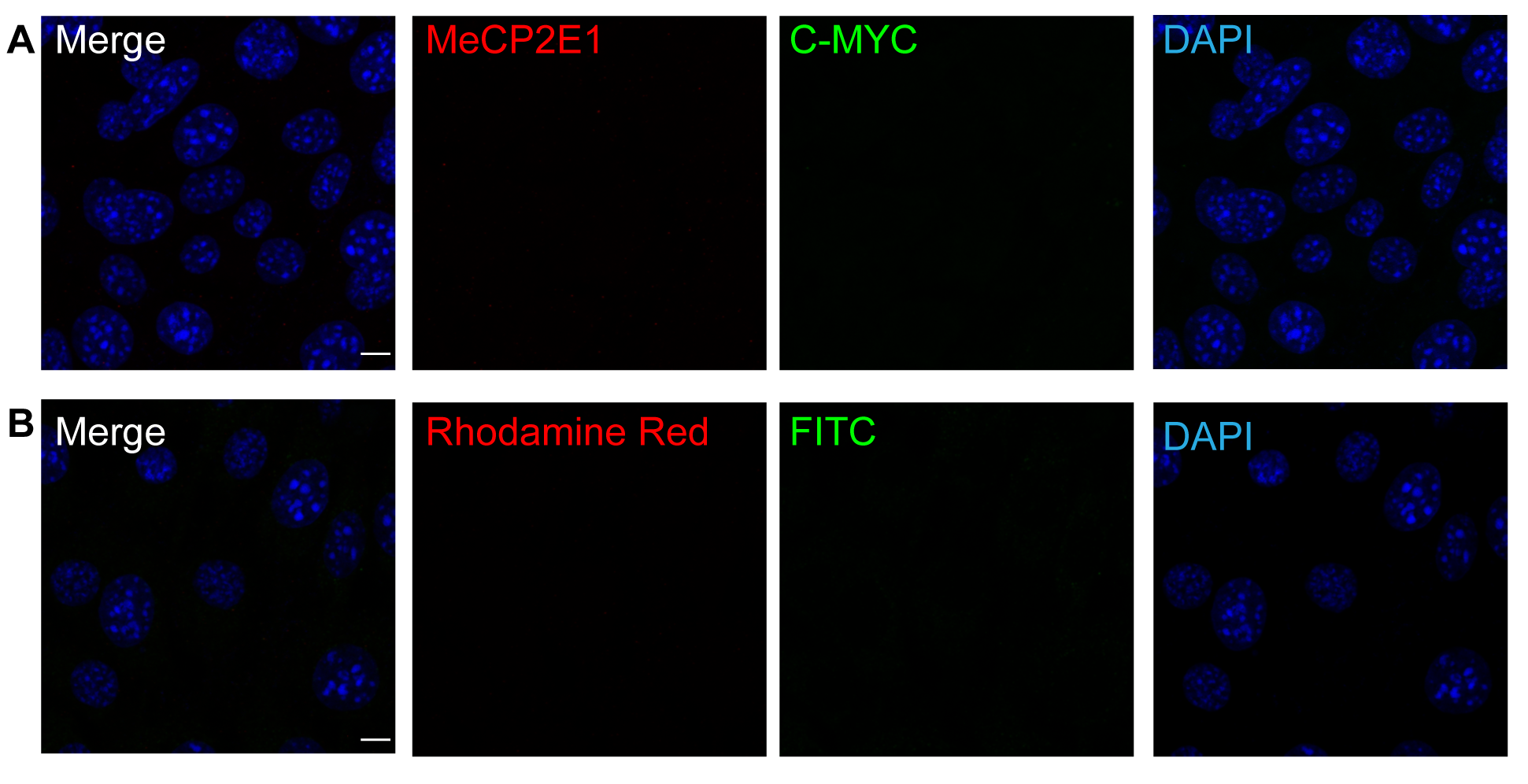

Supplement: Figure S1 — Controls for MeCP2 overexpression in NIH3T3 cells. A) Absence of MeCP2 and C-MYC signals in non-transfected NIH3T3 cells. B) Absence of signals in primary omission controls with Rhodamine Red and FITC in MECP2E1 transfected NIH3T3 cells. Images are taken at the same exposure time as in Figure 1E. Scale bars represent 10 µm. (TIFF) [file pone.0049763.s001.tiff]

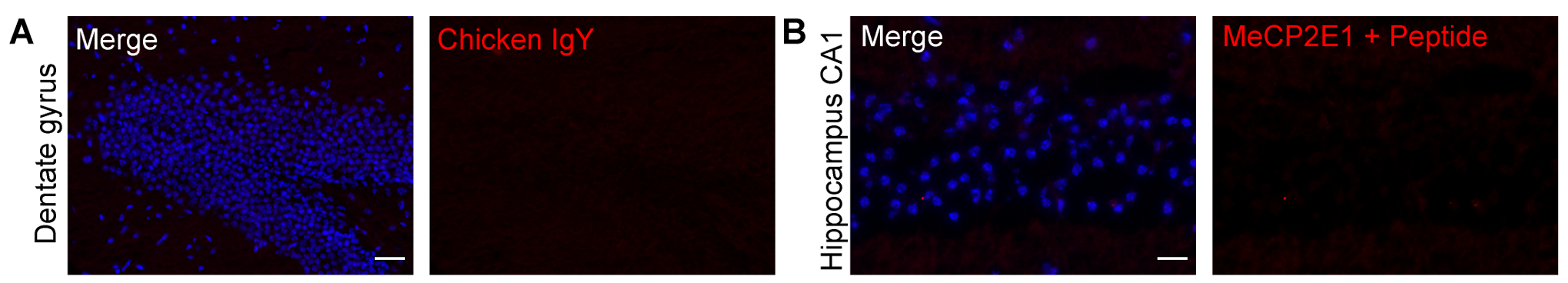

Supplement: Figure S2 — Controls to verify the specificity of MeCP2E1 immunolabelling within the adult murine brain. A) The negative control IgY did not generate any signals in Mecp2tm1.1Bird y/+ mice. B) Pre-incubation of the newly generated anti-MeCP2E1 with the antigenic peptide resulted in absence of specific labelling in Mecp2tm1.1Bird y/+ mice. Scale bars represent 20 µm. (TIFF) [file pone.0049763.s002.tiff]

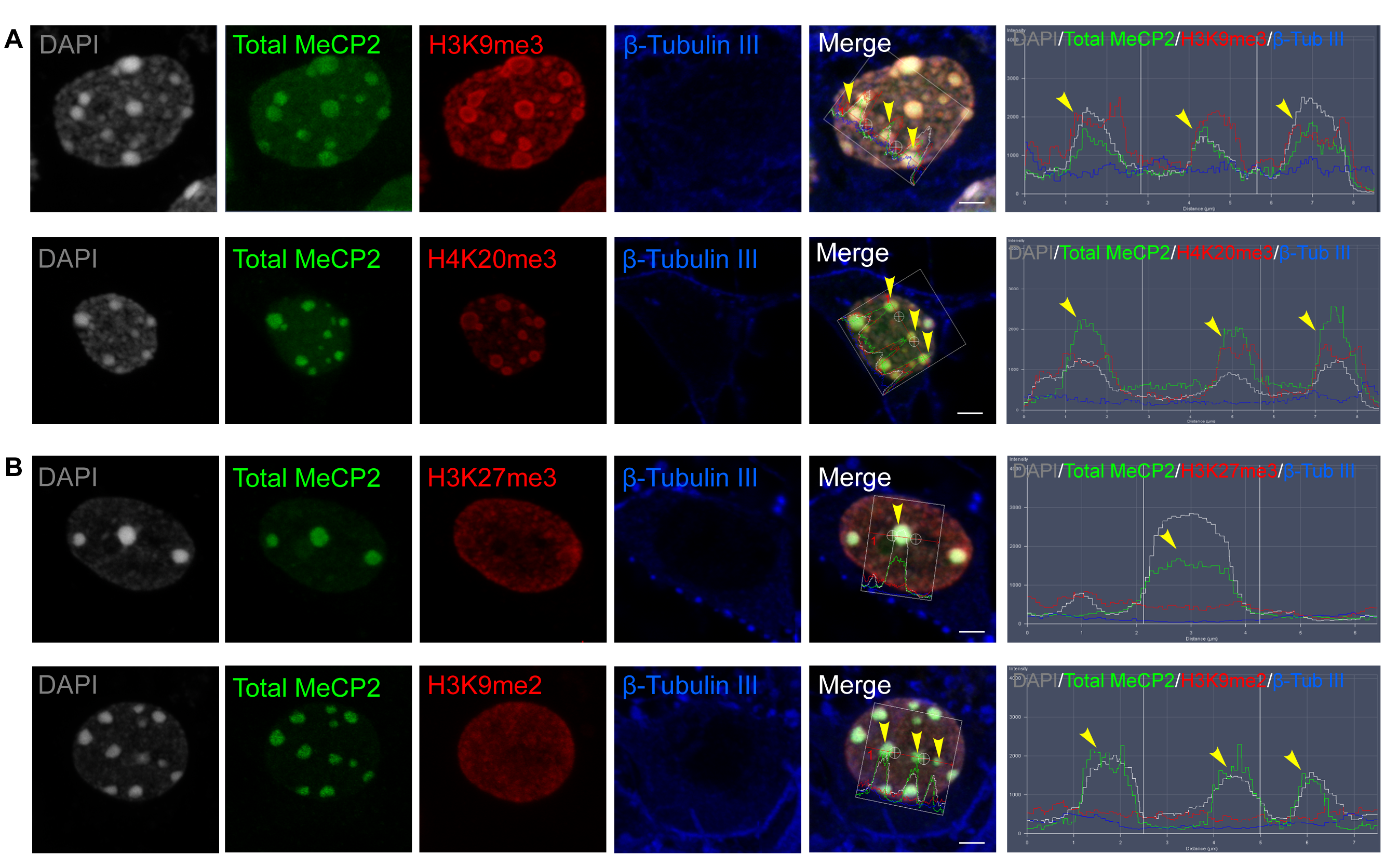

Supplement: Figure S3 — Nuclear localization of MeCP2 and heterochromatin marks in primary neurons. A) MeCP2 signals in embryonic primary cortical neurons display overlapped signals with constitutive heterochromatin marks; H3K9me3 and H4K20me3. B) MeCP2 displays minimal overlap with facultative heterochromatin marks; H3K27me3 and H3K9me2. Scale bars represent 2 µm. (TIFF) [file pone.0049763.s003.tiff]
